# Determinants of patient satisfaction in public hospitals and their remediabilities

**DOI:** 10.1186/1753-6561-6-S5-P4

**Published:** 2012-09-28

**Authors:** Nikhil Prakash Gupta, Parminder Gautam, JN Srivastava

**Affiliations:** 1 National Health Systems Resource Centre, New Delhi, India

## Introduction

Quality in healthcare consists of two aspects. While technical quality primarily deals with accuracy of diagnosis and procedures and adherence to clinical protocols, service quality refers to the manner in which the healthcare services are delivered to patients. Since patients are often unable to accurately assess the technical quality of care, functional/service quality is usually the primary determinant of patients’ perceptions of quality. This study tries to crystalise ‘critical to quality’ attributes of services from an ideal list of what should be there. We also explored the possible solutions for these issues that can be implemented in public health facilities.

## Methods

We developed questionnaires to assess patient satisfaction after understanding the ‘Voice of Customer’ (VOC) through field visits, interviews with patients and service providers and focus group discussions. Attributes distilled though VOC were arranged in an Ishikawa Diagram and converted into a 10-point questionnaire for outpatient and 20-point questionnaire for inpatient services. Patient perception was measured on a five-point Likert scale ranging from poor to excellent. We obtained feedback on this questionnaire on a quarterly basis from a sample size calculated for 95% confidence level. Data were analysed and lowest two indicators were identified for root-cause analysis and developing of an action plan. Data were further analysed on analytical hierarchy process model by pair-wise comparison to find out Critical to Quality (CTQs) factors. Relative weightage of CTQs were then analysed through Pareto analysis.

## Results

Data analysis of patient satisfaction survey across 9 district hospitals of Bihar shows that patients are most dissatisfied with non-availability of drugs and quantum of consultation time for outpatient care, while for inpatients, cleanliness of toilets mattered most. At the same time, non-availability of drugs is also the most dissatisfying factor for inpatients. Further, it emerges through pareto analysis that enhancement in patients’ satisfaction by more than 60% could be achieved by addressing top three attributes of dissatisfaction. Finally on Kano mode, non-availability of drugs and perception of not getting enough consultation time are ‘dissatisfiers’; cleanliness, ready availability of diagnostic services and prolonged waiting times as ‘performers’. Nice and courteous behaviour of staff of public hospitals are the ‘delighter’ for the patients.

## Discussion

Services unlike products are produced and consumed at the same time. This characteristic increases the importance of the care provider-patient relationship as well highlights potential for variation in service quality. With the advent of rights-based approach in health, patients are no more to be treated as targets or beneficiaries. They are customers or right seekers; service providers should try to meet their expectations. Patient satisfaction survey is one tool to measure, whether hospital services are meeting patients’ expectation or not. Patient satisfaction survey is an integral part of quality management system developed by the National Health Systems Resource Centre for public hospitals. These surveys help in improving the service quality if service providers can focus on the results of the surveys, which are critical to patients and plan their services accordingly. Quality function deployment is one such tool that can be used for planning and implementing solutions.

## Competing interests

None declared

## Funding statement

None declared

